# Association between heme oxygenase one and sepsis development in patients with moderate-to-critical COVID-19: a single-center, retrospective observational study

**DOI:** 10.1186/s40001-022-00915-5

**Published:** 2022-12-04

**Authors:** Hsin-Yi Chen, I-Shiang Tzeng, Kuo-Wang Tsai, Yao-Kuang Wu, Ching-Feng Cheng, Kuo-Cheng Lu, Hsueh-Wen Chung, You-Chen Chao, Wen-Lin Su

**Affiliations:** 1 Division of Pulmonary and Critical Care Medicine, Department of Internal Medicine, Taipei Tzu Chi Hospital, Buddhist Tzu Chi Medical Foundation, 289 Jianguo Rd, Xindian Dist., New Taipei City, 23142 Taiwan; 2 School of Medicine, Tzu Chi University, Hualien, 970 Taiwan; 3 Department of Medical Research, Taipei Tzu Chi Hospital, Buddhist Tzu Chi Medical Foundation, New Taipei City, Taiwan; 4 Department of Pediatrics, Taipei Tzu Chi Hospital, Buddhist Tzu Chi Medical Foundation, New Taipei City, 231 Taiwan; 5 Division of Nephrology, Department of Medicine, Taipei Tzu Chi Hospital, Buddhist Tzu Chi Medical Foundation, New Taipei, 231 Taiwan; 6 Department of Medicine, School of Medicine, College of Medicine, Fu-Jen Catholic University Hospital, Fu-Jen Catholic University, New Taipei City, 24205 Taiwan; 7 School of Nursing, National Yang Ming Chiao Tung University, Taipei, 112 Taiwan; 8 Department of Internal Medicine, Taipei Tzu Chi Hospital, Buddhist Tzu Chi Medical Foundation, New Taipei, 231 Taiwan

**Keywords:** Bilirubin, COVID-19, Ferritin, Heme, Heme oxygenase one, Sepsis

## Abstract

**Background:**

Heme oxygenase one (HO-1) is considered a poor prognostic factor for survival in patients with severe-to-critical coronavirus disease (COVID-19), but the clinical correlation between heme catabolism biomarkers and COVID-19-related sepsis is unknown. The etiopathogenetic hypothesis of HO-1 response during sepsis in patients with poor prognosis should be clarified. This study aimed to investigate sepsis development within 48 h following moderate-to-critical COVID-19 and examined heme/HO-1 catabolism biomarkers associated with sepsis. We also studied the HO-1 and traditional prognostic factors for predicting survival in patients with COVID-19.

**Methods:**

This retrospective observational study included patients unvaccinated for COVID-19 with moderate-to-critical COVID-19 (*n* = 156) who had been admitted to Taipei Tzu Chi Hospital in 2021. All COVID-19 patients were diagnosed by severe acute respiratory syndrome coronavirus 2 (SARS-CoV-2) reverse transcriptase polymerase chain reaction. For analysis of heme catabolism in SARS-CoV-2-induced sepsis, we excluded patients with co-infection and severe anemia. Heme catabolism biomarkers were compared between groups of patients with COVID-19 and sepsis (sepsis) and those with COVID-19 without sepsis (no sepsis), and a control group comprising 100 healthy individuals. All clinical and laboratory data were collected retrospectively and blood specimens were collected from Biobank. Multivariable logistic regression analysis was used to compare all variables between the sepsis and no-sepsis groups. Cox regression analysis was used to determine predictors of survival in patients with COVID-19.

**Results:**

There were 71 and 85 patients with and without sepsis, respectively. Heme and HO-1 levels differed significantly between the sepsis, no sepsis, and control groups. In multivariate analysis, confusion, blood urea nitrogen, respiration, blood pressure in patients aged > 65 years (CURB-65) (adjusted odds ratio [aOR] 5.331, 95% confidence interval [CI] 2.587–10.987; *p* < 0.001), albumin (aOR 0.139, 95% CI 0.003–0.636; *p* = 0.01), d-dimer (aOR 1.001, 95% CI 1.000–1.002; *p* = 0.032), and HO-1 (aOR 1.116, 95% CI 1.055–1.180; *p* < 0.001) were significantly associated with 48-h sepsis episodes after adjusting for other confounding factors. HO-1 levels were also significantly associated with 48-h Sequential Organ Failure Assessment Score (SOFA) scores. However, HO-1 did not significantly increase the hazard of in-hospital mortality in moderate-to-critical COVID-19 by Cox regression analysis.

**Conclusions:**

HO-1 levels increased with sepsis development within 48 h of admission for COVID-19 after adjusting for other risk factors, but no significant association was observed between HO-1 and COVID-19 mortality. We suppose that HO-1 may have protective effect in early sepsis, but further clinical multicenter prospective studies are needed.

## Background

Among patients with coronavirus disease (COVID-19) in China, only 5% became critically ill with complications, such as respiratory failure, septic shock, and multiple organ dysfunction or failure, but the mortality rate was high (49%) [1]. Detection of sepsis and septic shock is important for early treatment and to help prevent deteriorating clinical outcomes [2], especially in patients with COVID-19 [3].

Heme is an oxidant that can be degraded to carbon monoxide, ferritin, and bilirubin by heme oxygenase one (HO-1), and has been shown to play an essential antioxidant role in acute respiratory distress syndrome [4]. Carbon monoxide, ferritin, and bilirubin catabolites are known antioxidants. Hyperferritinemia [5] and hyperbilirubinemia [6] have been observed in critically ill patients, especially in those with sepsis and septic shock. One human sepsis study reported that high HO-1 levels were associated with sepsis severity and low survival [7]. Increased HO-1 expression has been found in severe-to-critically ill patients with COVID-19, with a poor survival prognosis [8]. However, the role of heme catabolism in COVID-19-related sepsis remains unknown. The etiopathogenetic hypothesis of HO-1 response during sepsis in patients with poor prognosis should be clarified. This study aimed to compare differences in heme catabolism, including HO-1 activity, between patients with and without sepsis development within 48 h of a COVID-19-related admission. We also evaluated the HO-1 and traditional prognostic factors for predicting survival in patients with COVID-19.

## Methods

### Study design and enrollment

Patients with COVID-19 were enrolled retrospectively during the COVID-19 pandemic (from May 1 to October 31, 2021) at Taipei Tzu Chi Hospital, Taiwan. We included patients with at least moderate COVID-19 and evidence of pneumonia on chest radiography. Reverse transcriptase polymerase chain reaction (RT-PCR) tests for severe acute respiratory syndrome coronavirus 2 (SARS-CoV-2) were used to diagnose COVID-19. Patients were registered in the COVID-19 biobank of Taipei Tzu Chi Hospital during the pandemic in 2021. To determine HO-1 catabolism associated with sepsis development in patients with COVID-19, we excluded the following patients: (i) those who had co-infection with other bacteria within 48 h of admission; (ii) those who had blood specimens collected 2 days after admission, as this might have influenced the association analysis, and (iii) those with severe anemia (hemoglobin, < 8 g/dL), as this may have influenced heme catabolism during sepsis.

**Fig. 1:**
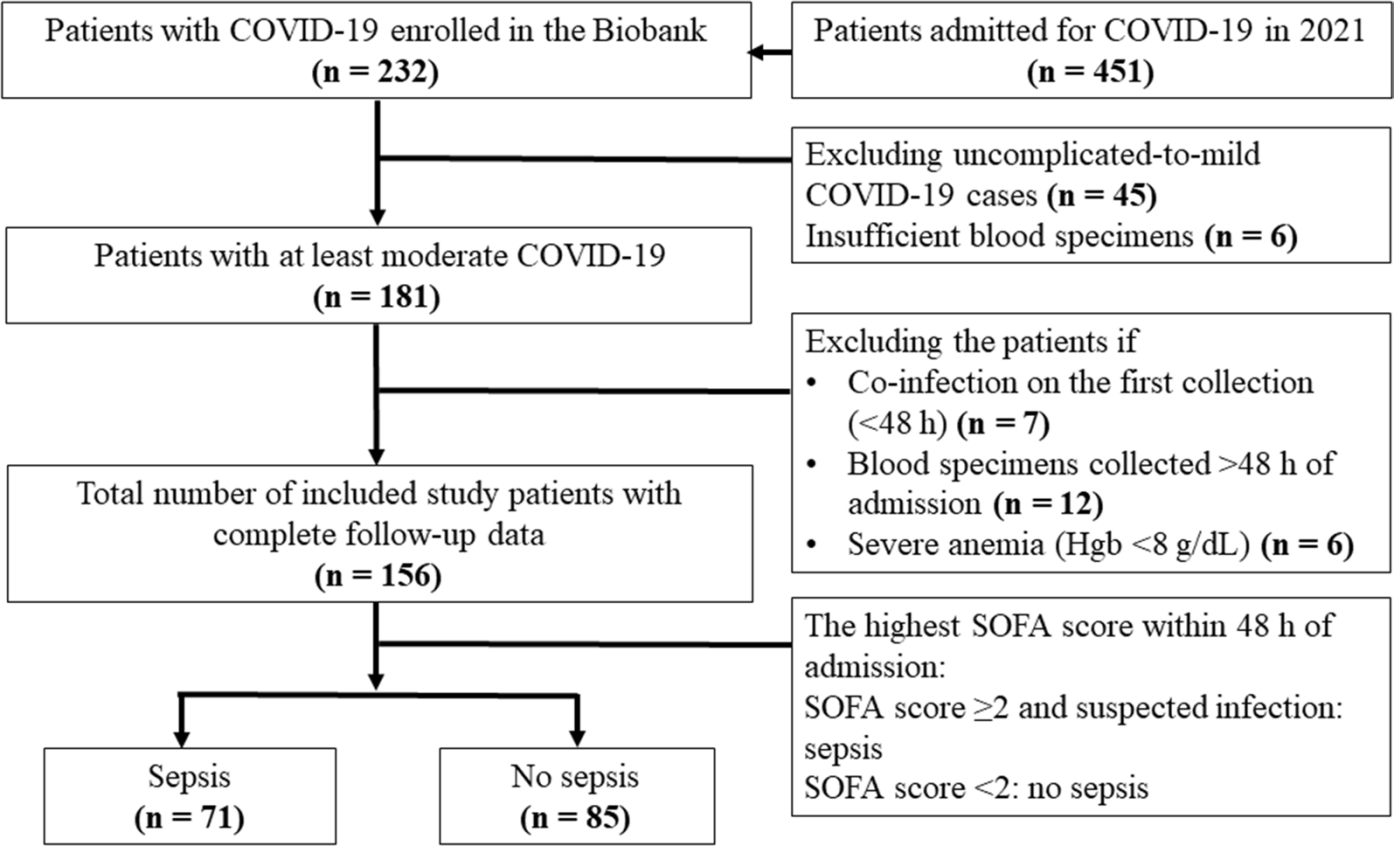
Flow diagram of the study enrollment process. A total of 451 patients diagnosed with COVID-19 were admitted to Taipei Tzu Chi Hospital during the 2021 pandemic. During the pandemic, the basic data and blood samples of patients who received oxygen in the ICU and quarantine ward were collected and stored in the Biobank of Taipei Tzu Chi Hospital, Buddhist Tzu Chi Medical Foundation, and informed consent was obtained. A COVID-19 blood sample biobank was set up for storage during the 2021 pandemic. Of 451 patients, basic data of 232 patients with COVID-19 from the Biobank were retrieved and included in this retrospective cohort study, which was approved by the Institutional Review Board (IRB) of Taipei Tzu Chi Hospital, Buddhist Tzu Chi Medical Foundation. After screening the patients’ electronic medical records, 45 patients with uncomplicated-to-mild COVID-19 were excluded owing to zero risk of developing sepsis until discharge from the hospital. Blood specimens were insufficient in six patients. A total of 181 patients with COVID-19 of at least moderate severity were examined for eligibility. To determine the associations of sepsis, seven and 12 patients who had co-infection or had no blood specimens within 48 h after admission, respectively, and six patients with severe anemia were excluded. Finally, 156 patients who had not previously received vaccinations were enrolled in the study

In total, 451 patients infected with COVID-19 were identified and screened. After applying the inclusion and exclusion criteria, 156 patients were included in the study (Fig. 1). A control group comprising 100 healthy volunteers whose blood samples had been collected and stored in the Taipei Tzu Chi Hospital biobank following informed consent, were also enrolled.

### Ethics approval and consent to participate

This single-center, retrospective cohort study was approved by the Institutional Review Board of Taipei Tzu Chi Hospital, Buddhist Tzu Chi Medical Foundation, and New Taipei City, Taiwan, on August 10, 2022 (Protocol No.:11-X-112). This study was conducted in accordance with the guidelines of the amended Declaration of Helsinki. The study protocol was reviewed, and an informed consent waiver was obtained from the Institutional Review Board. The application of bio-specimens in this study was reviewed and approved by the Biobank Ethics Committee (Application No.: Tzubiobank 2022-01).

### Disease definition and treatment

National Institute of Health guidelines [9] define moderate COVID-19 as a combination of pneumonia and not life-threatening symptoms, such as cough with expectoration, fever, general body pain, and weakness; oxygen saturation of pulse oximeter (SpO_2_) ≥ 94% in room air. Severe COVID-19 is defined as clinical signs of pneumonia (fever, cough, dyspnea, rapid breathing) and ≥ 1 of the following symptoms: respiratory rate, > 30 breaths/min; severe respiratory distress; ratio of the partial pressure of oxygen in the arterial blood to the fraction of inspired oxygen, < 300; SpO_2_ < 94% in room air; or infiltration, > 50%. Patients with critical COVID-19 were confirmed to have SARS-CoV-2 infection and were admitted to the intensive care unit for respiratory and/or cardiovascular organ support, including those with acute respiratory distress syndrome, respiratory failure requiring ventilation, sepsis, and/or septic shock.

An acute change in the total Sequential Organ Failure Assessment Score (acute SOFA changes ≥ 2) attributable to infection was calculated retrospectively to define sepsis diagnosis within 48 h of admission [10, 11]. Septic shock was defined as sepsis with persisting hypotension requiring vasopressors to maintain mean arterial pressure (MAP) ≥ 65 mmHg and a serum lactate level > 2 mmol/L (18 mg/dL) despite adequate volume resuscitation [10]. We also collected data in relation to multiple organ dysfunction syndrome/multiple organ failure within 48 h of admission.

In Taiwan, remdesivir and dexamethasone were available for patients with moderate-to-critical COVID-19 during the pandemic in 2021. Treatment was applied in accordance with Taiwan Centers for Disease Control guidance and recommendations by Hsueh et al. [12] Surviving Sepsis campaign guidelines on the management of patients with COVID-19 were followed when treating patients with sepsis and septic shock [3].

### Measurements

Demographic characteristics, such as age, sex, cycle threshold value (CtV) of SARS-CoV-2 based on RT-PCR test results, body mass index (BMI), initial body temperature, respiratory rate, heart rate, SpO_2_ level, Glasgow Coma Scale (GCS) score, previous use of anticoagulants or anti-platelets, confusion, blood urea nitrogen, respiration, blood pressure in those aged > 65 years (CURB-65) [13], quick SOFA score [10], and Charlson comorbidity index (CCI) score [14], were collected for analysis. The initial CURB-65 score at admission was measured retrospectively based on the presence of confusion (newly disoriented in person, place, or time), blood urea nitrogen (BUN) level > 20 mg/dL, respiratory rate ≥ 30 breaths/min, blood pressure (systolic [SBP]) < 90 mmHg or diastolic blood pressure (≤ 60 mmHg), and age ≥ 65 years. The quick SOFA (qSOFA) score was calculated by adding one point for any of the following criteria: altered mental status (GCS < 15), high respiratory rate (≥ 22/min), or low blood pressure (SBP ≤ 100 mmHg). Additionally, underlying comorbidities (chronic neurologic disease [disabling neurologic conditions], chronic lung disease [chronic obstructive or restrictive lung disease], chronic cardiovascular disease [coronary atherosclerotic disease and congestive heart failure], chronic liver disease [liver cirrhosis], chronic kidney disease [end-stage renal disease], diabetes mellitus, cancer, and autoimmune diseases) were retrospectively recorded and used to derive CCI scores retrospectively.

The results of blood tests, including complete blood count, biochemistry, C-reactive protein (CRP), total bilirubin, ferritin, and d-dimer levels, were also collected retrospectively.

### Heme and HO-1 enzyme-linked immunosorbent assay (ELISA) analysis

All serum/plasma samples were obtained from the biobank and stored at − 80 °C until analysis. Double-antibody sandwich enzyme immunoassay assays were developed for HO-1 (Cat No. ab215401), and an improved aqueous alkaline solution method was used for heme analysis (Cat. ab272534). All items were evaluated according to the manufacturer’s guidelines, as described in the following and previous descriptions [15].

### Manufacturer’s instructions for HO-1 ELISA kit

According to the manufacturer’s instructions, a 96-well plate was coated with capture antibodies overnight. Subsequently, a wash buffer was used to remove excess antibodies. A blocking buffer was used overnight to block the voids, and the plate was washed again to complete the capture-antibody coating step. Test samples and standards were transferred to a coated 96-well plate, assessed in duplicate, and incubated at room temperature (RT) for 2 h. This was followed by subsequent washing of the plate and addition of a diluted detection antibody (0.5 μg/mL). After incubation at RT for 1 h, the plates were washed again. This was followed by the addition of streptavidin–horseradish peroxidase solution, incubation at RT for 1 h, and subsequent washing of the plate. Next, the TMB substrate was added and incubated at RT for 5–10 min (care was taken to avoid light exposure). In the final step, a stop solution was used to terminate the reaction, and the absorbance was measured at 450 nm as the reference wavelength.

### Manufacturer’s instructions for the heme assay kit

As per the instructions, the calibrator and each sample were added to the appropriate wells, and ddH_2_O was added to the blank wells. Bubble formation was avoided during the addition step. ddH_2_O was added to the blank and calibrator wells (this chroma was the same as 62.5 μM heme). Detect reagent was added to each sample well, incubated at room temperature for 5 min, and measured at OD380–420 nm as the reference wavelength.

### Clinical outcomes

The clinical outcomes in this study were sepsis development at 48 h of admission, organ dysfunction/failure, septic shock, the 48-h SOFA score, survival, final COVID-19 severity, and total hospitalization days.

### Statistical analysis

Analysis of variance was used for comparisons among the three groups (sepsis vs. no sepsis vs. control groups). Additionally, we performed a post hoc analysis (Scheffe’s test) for continuous variables that showed significant differences. Continuous data are expressed as mean ± standard deviation. Categorical data are expressed as frequencies and percentages. Demographic and clinical characteristics were compared using a Student’s *t-*test between the sepsis and no-sepsis groups, and a Chi-square test was used to compare the categorical variables. Multivariate analysis using logistic regression was performed to determine heme catabolism factors associated with 48-h sepsis episodes after adjusting for confounding factors. Multiple linear regression was performed to determine heme catabolism factors associated with 48-h SOFA scores after adjusting for confounding factors. Data were analyzed using IBM SPSS Statistics for Windows (version 24.0; IBM Corp, Armonk, NY, USA) software, and a *p*-value of < 0.05 was considered statistically significant.

## Results

### Heme catabolic biomarker levels among sepsis, no sepsis, and control groups

**Fig. 2:**
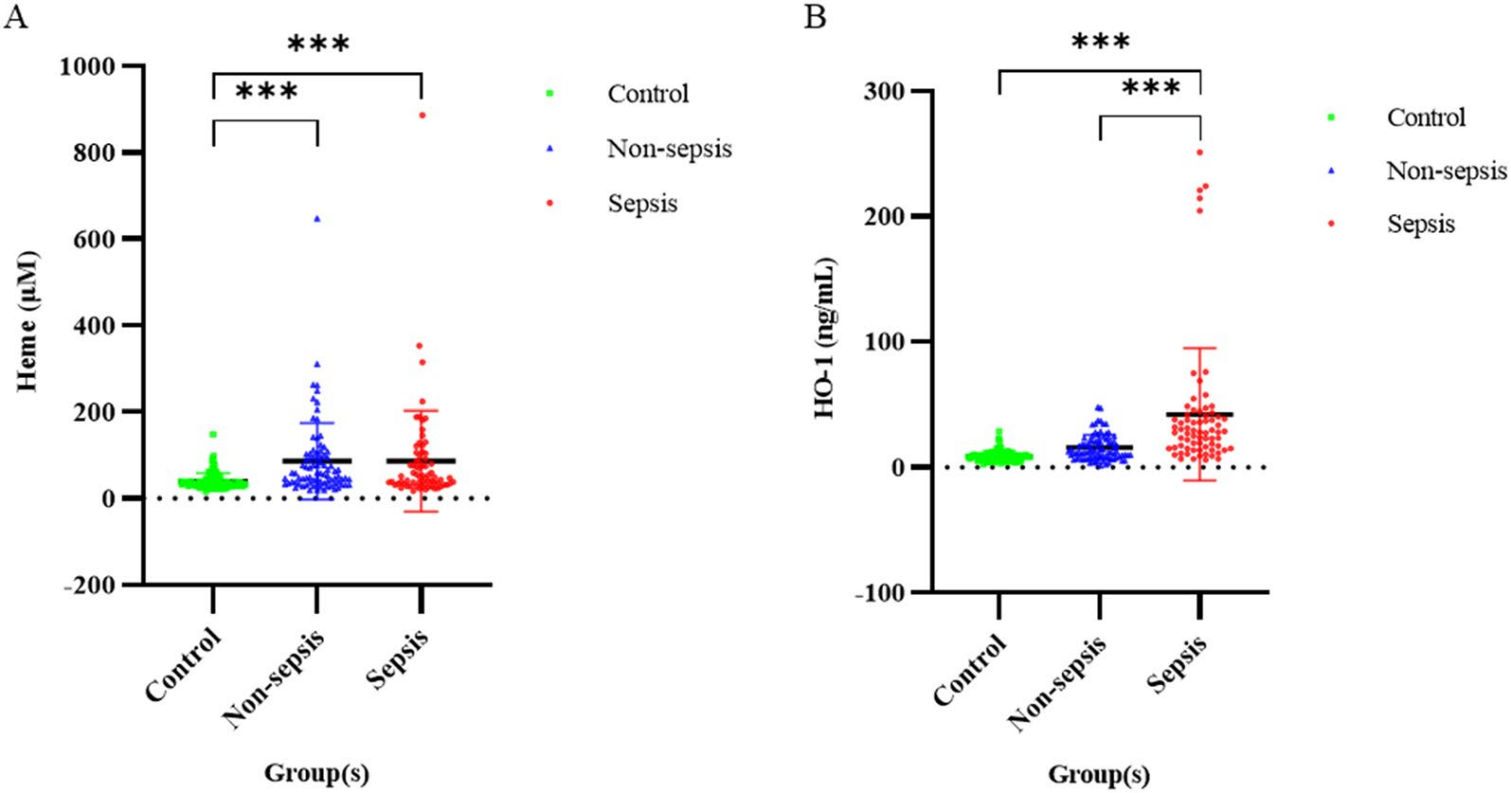
Influence of coronavirus disease (COVID-19) infection on patients' heme catabolism. **A** Heme and **B** HO-1. *p*-values calculated using a Student’s *t*-test are shown above the scatter points. ***Indicates *p* < 0.001 for the difference between paired scatter points, respectively

We divided 156 patients with COVID-19 of at least moderate severity into the following groups: sepsis (*n* = 71), no sepsis (*n* = 85), and healthy controls (*n* = 100). Heme values in the sepsis, no sepsis, and control groups were 86.3 ± 116.5, 86.2 ± 88.3, and 39.7 ± 19.2 µM, respectively; *p* < 0.001 (Fig. 2). A post hoc analysis indicated that heme values were significantly higher in the sepsis and no-sepsis groups compared with the control group (*p* < 0.001). HO-1 values in the sepsis, no sepsis, and control groups were 39.9 ± 53.0, 17.7 ± 13.0, and 8.8 ± 4.2 ng/mL, respectively; *p* < 0.001. In a post hoc analysis for HO-1, the sepsis group had significantly higher values than those observed in the no sepsis and control groups (*p* < 0.001). Heme and HO-1 levels differed significantly between patients with COVID-19 and those in the control group.

Baseline characteristics are shown in Table 1. No significant differences were found between the sepsis and no-sepsis groups in terms of SARS-CoV-2 CtV, BMI, initial body temperature, respiratory rate, heart rate, GCS, diabetes mellitus, neurologic disease, chronic kidney disease, chronic liver disease, malignancy, and autoimmune disease. However, there were significant differences between the sepsis and no-sepsis groups regarding older age (64.7 vs. 54.7 years, *p* < 0.001), male sex (62.0% vs. 43.5%, *p* = 0.022), lower initial mean MAP (90.3 vs. 97.3 mmHg, *p* = 0.002), SpO2% (90.5% vs. 94.4%, *p* = 0.002), higher CCI (3.13 vs. 2.12, *p* = 0.001), higher cardiovascular disease comorbidity (35.2% vs. 16.5%, *p* = 0.007), higher pulmonary disease comorbidity (12.7% vs. 3.5%, *p* = 0.039), higher initial qSOFA (0.41 vs. 0.14, *p* = 0.002), and higher initial CURB-65 score (1.82 vs. 0.45, *p* < 0.001), respectively.

**Table 1:** Comparison of study participant characteristics (*n* = 156)

Variables	Sepsis (*n* = 71)	No sepsis (*n* = 85)	*p*-value
SARS-CoV-2 CtV	23.11 ± 6.07	23.05 ± 6.47	0.948^c^
Age (years)	64.72 ± 13.20	54.66 ± 15.15	< 0.001**^c^
Sex			0.022*^a^
Female	27 (38.0%)	48 (56.5%)	
Male	44 (62.0%)	37 (43.5%)	
Body mass index (m/kg^2^)	26.47 ± 5.60	25.11 ± 3.79	0.073*^c^
Vital signs			
Body temperature (°C)	37.27 ± 1.14	37.10 ± 1.04	0.332^c^
Respiratory rate (/min)	19.65 ± 4.80	18.87 ± 1.44	0.192^c^
Heart rate (/min)	91.27 ± 20.14	89.88 ± 16.01	0.633^c^
MAP (mmHg)	90.27 ± 14.19	97.32 ± 13.65	0.002*^c^
SpO_2_ (%)	90.50 ± 10.00	94.35 ± 1.79	0.002*^c^
GCS	14.90 ± 0.45	14.99 ± 0.11	0.118^c^
CCI	3.13 ± 2.04	2.12 ± 1.65	0.001*^c^
Comorbidities			
Diabetes	21 (29.6%)	18 (21.2%)	0.228^a^
Neurologic diseases	6 (8.5%)	7 (8.2%)	0.961^a^
Cardiovascular disease	25 (35.2%)	14 (16.5%)	0.007*^a^
Pulmonary disease	9 (12.7%)	3 (3.5%)	0.039*^b^
Chronic kidney disease	1 (1.4%)	0 (0%)	0.455^b^
Chronic liver disease	2 (2.8%)	5 (5.9%)	0.456^b^
Malignancy	2 (2.8%)	1 (1.2%)	0.592^b^
Autoimmune disease	1 (1.4%)	0 (0%)	0.455^b^
qSOFA	0.41 ± 0.65	0.14 ± 0.35	0.002*^c^
Initial CURB-65	1.82 ± 1.07	0.45 ± 0.57	< 0.001**^c^

*CCI* Charlson comorbidity index, *CtV* cycle threshold value, *CURB-65* confusion, blood urea nitrogen, respiration, blood pressure, in patients aged > 65 years, *GCS* Glasgow Coma Scale, *MAP* mean arterial pressure, *SpO*_*2*_ saturation from pulse oximeter, *qSOFA* quick Sequential Organ Failure Assessment

*p < 0.05; **p < 0.001

^a^A Chi-square test was used for the comparison analysis

^b^Fisher’s exact test was used for the comparison analysis

^c^An independent *t*-test was used for continuous variables between the sepsis and no-sepsis groups

### Laboratory data

Comparisons of blood test results between patients in the sepsis and no-sepsis groups are shown in Table 2. No differences in red blood cell count, or in hemoglobin, monocyte, serum glutamic pyruvic transaminase, alkaline phosphatase, creatinine phosphokinase, prothrombin time, international normalized ratio, activated partial thromboplastin clotting time (aPTT), heme, or total bilirubin levels were observed between the sepsis and no-sepsis groups. However, comparison of the sepsis group with the no-sepsis group showed that the sepsis group higher white blood cell (7.78 vs. 6.05 10^3^/µL, *p* = 0.004); lower platelet (196.8 vs. 248.2 10^3^/µL, *p* = 0.001), higher neutrophil (6.40 vs. 4.13 10^3^/µL, *p* < 0.001), and lower lymphocyte (0.80 vs. 1.43 10^3^/µL, *p* < 0.001) counts, and lower sodium (135.1 vs. 136.9 mEq/L, *p* = 0.002), higher potassium (3.9 vs. 3.7 mEq/L, *p* = 0.028), higher serum glutamic oxaloacetic transaminase (SGOT) (41.9 vs. 29.3 U/L, *p* = 0.004), lower albumin (3.39 vs. 3.91 g/dL, *p* < 0.001), higher BUN (26.1 vs. 13.3 mg/dL, *p* < 0.001), higher creatinine (1.42 vs. 0.76 mg/dL, *p* = 0.017), higher random glucose (170.5 vs. 124.2 mg/dL, *p* = 0.001), higher CRP (8.46 vs. 3.12 mg/dL, *p* < 0.001), higher lactate dehydrogenase (442.0 vs. 251.8 U/L, *p* < 0.001), and higher d-dimer (3648.0 vs. 631.7, *p* < 0.001) levels. In comparison, concerning HO-1 catabolism biomarkers, including HO-1, those in the sepsis group had higher HO-1 catabolism biomarkers than those in the no-sepsis group (42.1 vs. 15.8 ng/mL, respectively; *p* < 0.001) and ferritin (752.4 vs. 493.6 ng/mL, respectively; *p* = 0.006) (Fig. 2, Table 2). However, no significant differences were observed in terms of heme and total bilirubin levels between the two groups.

**Table 2:** A comparison of blood test results in patients with COVID-19 (*n* = 156)

Variable	Sepsis (*n* = 71)	No sepsis (*n* = 85)	*p*-value
WBC (10^3^/µL)	7.78 ± 4.55	6.05 ± 1.97	0.004*
RBC (10^6^/µL)	4.48 ± 0.74	4.51 ± 0.62	0.782
Hemoglobin (g/dL)	13.23 ± 2.01	13.44 ± 1.88	0.513
Platelets (10^3^/µL)	196.76 ± 97.21	248.18 ± 93.20	0.001*
Neutrophil (10^3^/µL)	6.40 ± 4.32	4.13 ± 1.68	< 0.001**
Lymphocyte (10^3^/µL)	0.80 ± 0.44	1.43 ± 0.75	< 0.001**
Monocyte (10^3^/µL)	0.42 ± 0.32	0.40 ± 0.21	0.645
Na (mEq/L)	135.13 ± 3.68	136.94 ± 3.57	0.002*
K (mEq/L)	3.90 ± 0.68	3.69 ± 0.44	0.028*
SGOT (U/L)	41.85 ± 26.72	29.27 ± 26.79	0.004*
SGPT (U/L)	32.93 ± 23.42	29.45 ± 24.15	0.365
ALP (U/L)	65.34 ± 28.96	57.65 ± 26.63	0.089
Albumin (g/dL)	3.39 ± 0.48	3.91 ± 0.43	< 0.001**
BUN (mg/dL)	26.06 ± 21.31	13.29 ± 4.21	< 0.001**
Creatinine (mg/dL)	1.42 ± 2.29	0.76 ± 0.22	0.017*
Random glucose (mg/dL)	170.54 ± 103.34	124.15 ± 52.32	0.001*
CPK (U/L)	189.20 ± 346.16	109.94 ± 100.20	0.066
CRP (mg/dL)	8.46 ± 6.87	3.12 ± 4.99	< 0.001**
LDH (U/L)	416.51 ± 197.47	246.94 ± 103.70	< 0.001**
D-dimer (ng/mL)	3648.02 ± 3746.70	631.65 ± 444.75	< 0.001**
PT (s)	11.93 ± 8.68	10.51 ± 0.50	0.172
INR (ratio)	1.17 ± 1.03	1.01 ± 0.05	0.172
aPTT (s)	30.53 ± 6.77	29.48 ± 3.36	0.234
HO-1 catabolism			
Heme (μM)	86.34 ± 116.47	86.23 ± 88.26	0.995
HO-1 (ng/mL)	42.13 ± 52.72	15.82 ± 10.15	< 0.001**
Total bilirubin (mg/dL)	0.70 ± 0.38	0.62 ± 0.25	0.118
Ferritin (ng/mL)	752.44 ± 493.14	493.63 ± 648.90	0.006*

An independent *t*-test was used. *p < 0.05; **p < 0.001

*ALP* alkaline phosphatase, *aPTT* activated partial thromboplastin time, *BUN* blood urea nitrogen, *COVID-19* coronavirus disease, *CPK* creatine phosphokinase, *CRP* C-reactive protein, *HO-1* heme oxygenase one, *INR* international normalized ratio, *LDH* lactate dehydrogenase, *PT* prothrombin time, *SGOT* serum glutamic-oxaloacetic transaminase, *SGPT* serum glutamate pyruvate transaminase, *WBC* white blood count

### Clinical outcomes

Patients in the sepsis group had poorer clinical outcomes than those in the no-sepsis group (Table 3). Based on the final COVID-19 severity definition, those in the sepsis group had significantly worse critical illness compared with those in the no-sepsis group (78.9% vs. 0%, *p* < 0.001), more hospitalization days (27.5 vs. 16.2, *p* < 0.001), greater circulation dysfunction (12.7% vs. 0%, *p* = 0.001), greater respiratory dysfunction (81.7% vs. 0%, *p* < 0.001), greater renal dysfunction (29.6% vs. 2.4%, *p* < 0.001), greater hematologic dysfunction (39.4% vs. 14.1%, *p* < 0.001), greater septic shock (8.5% vs. 0%, *p* = 0.008), higher 48-h SOFA scores (4.28 vs. 0.21, *p* < 0.001), and lower survival rates (64.8% vs. 100.0%, *p* < 0.001), respectively.

**Table 3:** A comparison of the clinical outcomes between sepsis and no-sepsis groups (*n* = 156)

Variables	Sepsis (*n* = 71)	No sepsis (*n* = 85)	*p*-value
Clinical outcomes			
Severity of COVID-19			< 0.001**^a^
Moderate illness	4 (5.6%)	55 (64.7%)	
Severe illness	11 (15.5%)	30 (35.3%)	
Critical illness	56 (78.9%)	0 (0.0%)	
Hospital days	27.54 ± 19.53	16.02 ± 7.78	< 0.001**^c^
Organ dysfunction			
Circulation dysfunction	9 (12.7%)	0 (0%)	0.001*^b^
Hepatobiliary dysfunction	7 (9.9%)	3 (3.5%)	0.187^b^
Neurologic dysfunction	4 (5.6%)	1 (1.2%)	0.178^b^
Respiratory dysfunction	58 (81.7%)	0 (0%)	< 0.001**^b^
Renal dysfunction	21 (29.6%)	2 (2.4%)	< 0.001**^b^
Hematologic dysfunction	28 (39.4%)	12 (14.1%)	< 0.001**^a^
Septic shock	6 (8.5%)	0 (0%)	0.008*^b^
48-h SOFA score	4.28 ± 1.86	0.21 ± 0.41	< 0.001**^c^
Survival	46 (64.8%)	85 (100.0%)	< 0.001**^a^

*COVID-19* coronavirus disease, *SOFA* Sequential Organ Failure Assessment

*p < 0.05; **p < 0.001

^a^A Chi-square test was used for comparison analysis

^b^Fisher’s exact test was used for comparison analysis

^c^An independent *t*-test was used for continuous variables between sepsis and no-sepsis groups

### Factors associated with 48-h sepsis in patients with at least moderate COVID-19-related pneumonia

The multivariable logistic regression analysis was forward conditionally adjusted for all the variables with *p* < 0.05 in the comparisons between the sepsis and no-sepsis groups. The adjusted factors were initial CURB-65 (adjusted odds ratio [aOR] 5.331, 95% confidence interval [CI] 2.587–10.987; *p* < 0.001), albumin (aOR 0.139, 95% CI 0.003–0.636; *p* = 0.01), d-dimer (aOR 1.001, 95% CI 1.000–1.002; *p* = 0.032), and HO-1 (aOR 1.116, 95% CI 1.055–1.180; *p* < 0.001) levels; these factors were significantly associated with 48-h sepsis episodes (Table 4).

**Table 4:** Logistic regression models of sepsis factors (*n* = 156)

Variables	$\beta$	SE	OR	95% CI		*p*-value
				Lower	Upper	
Initial CURB-65	1.674	0.369	5.331	2.587	10.987	< 0.001**
Albumin (g/dL)	− 1.977	0.777	0.139	0.030	0.636	0.011*
d-dimer (ng/mL)	0.001	0.000	1.001	1.000	1.002	0.032*
HO-1 (ng/mL)	0.109	0.029	1.116	1.055	1.180	< 0.001**

Logistic regression model with forward conditional method selection

*CI* confidence interval, *CURB-65* confusion; blood urea nitrogen, respiratory rate, blood pressure, in patients aged > 65 years; HO-1, heme oxygenase-1; OR, odds ratio; SE, standard error

*p < 0.05; **p < 0.001

Furthermore, the multivariable linear regression analysis was adjusted stepwise for all independent variables (*p* < 0.05) to predict the dependent variable 48-h SOFA scores. Finally, initial CURB-65, d-dimer, aPTT, BMI, creatinine, SpO_2_, CRP, and HO-1 levels were significantly associated with the 48-h SOFA score, as shown in Table 5.

**Table 5:** Multiple linear regression models of 48-h SOFA score factors (*n* = 156)

Variables	$\beta$	SE	$\beta$ 95% CI		*p*-value
			Lower	Upper	
Initial CURB-65	0.582	0.119	0.347	0.817	< 0.001**
d-dimer (ng/mL)	0.323 × 10^–3^	0.044 × 10^–3^	0.236 × 10^–3^	0.411 × 10^–3^	< 0.001**
aPTT (s)	0.084	0.022	0.039	0.128	< 0.001**
BMI (m/kg^2^)	0.093	0.024	0.046	0.139	< 0.001**
Creatinine (mg/dL)	0.282	0.073	0.137	0.426	< 0.001**
SpO_2_ (%)	-0.051	0.017	-0.085	-0.017	0.004*
CRP (mg/dL)	0.062	0.019	0.023	0.100	0.002*
HO-1 (ng/mL)	0.016	0.003	0.010	0.022	< 0.001**

Multiple linear regression model: stepwise method selection

*aPTT* activated partial thromboplastin time, *BMI* body mass index, *CI* confidence interval, *CRP* C-reactive protein, *CURB-65* confusion; blood urea nitrogen, respiratory rate, blood pressure, in patients aged > 65 years, *HO-1* heme oxygenase-1, *OR* odds ratio, *SE* standard error, *SOFA* Sequential Organ Failure Assessment, *SpO*_*2*_ saturation of pulse oximeter

*p < 0.05; **p < 0.001. Dependent variable: SOFA score

In the sepsis subgroup analysis, those who survived had significantly lower HO-1 levels than those who did not survive (30.5 vs. 63.5 ng/mL, respectively; *p* = 0.042). There was no significant difference between patients who survived and those who did not in terms of heme, total bilirubin, and ferritin levels. Further evaluation in the multivariable Cox regression analysis (Table 6) showed that CCI [adjusted hazard ratio (aHR) = 1.659, 95% CI 1.283–2.145, *p* < 0.001], initial CURB-65 [aHR = 2.000, 95% CI 1.238–3.231, *p* = 0.005], and 48-h SOFA score [aHR = 1.730, 95% CI 1.353–2.210, *p* < 0.001] increased the hazard of in-hospital mortality. However, HO-1 did not significantly increase the hazard of in-hospital mortality in moderate-to-critical COVID-19.

**Table 6:** Cox regression model after adjusting for other confounding factors

	Bivariate		Multivariate	
	HR (95% CI)	*p*-value	HR (95% CI)	*p*-value
SARS-CoV-2 CtV	0.971 (0.909–1.039)	NS		
Sex	1.282 (0.575–2.858)	NS		
Body mass index	1.013 (0.947–1.084)	NS		
CCI	1.485 (1.258–1.752)	< 0.001*	1.659 (1.283–2.145)	< 0.001*
qSOFA	1.473 (0.880–2.465)	NS		
Initial CURB-65	2.518 (1.702–3.727)	< 0.001*	2.000 (1.238–3.231)	0.005*
SOFA score	1.532 (1.284–1.827)	< 0.001*	1.730 (1.353–2.210)	< 0.001*
HO-1 catabolism				
Heme	0.997 (0.991–1.003)	NS		
HO-1	1.009 (1.004–1.014)	0.001*	1.001 (0.995–1.007)	NS
Total bilirubin	2.254 (0.978–5.198)	NS		
Ferritin	1.000 (1.000–1.001)	NS		

Events: in-hospital mortality, time: days since ED admission to discharge or in-hospital mortality

SOFA score was calculated within the 48 h of admission

Selection: stepwise

*CCI* Charlson comorbidity index, *CI* confidence interval, *CURB-65* confusion; blood urea nitrogen, respiratory rate, blood pressure, in patients aged > 65 years, *CtV* cycle threshold value, *ED* emergency department, *HO-1* heme oxygenase-1, *HR* hazard ratio, *NS* non-significant, *qSOFA* quick Sepsis-Related Organ Failure Assessment, *SARS-CoV-2* severe acute respiratory syndrome-corona virus-2

(*) Statistical significant (*p* < 0.05)

## Discussion

This study aimed to investigate the association between heme catabolism biomarkers, such as HO-1, and subsequent sepsis development within 48 h of admission in patients with moderate-to-critical COVID-19-related pneumonia, particularly in unvaccinated COVID-19 populations.

In our previous study, heme and HO-1 levels were found to increase with low saturation in patients with COVID-19 [15]. Heme related to hemolysis drove poor sepsis-related outcomes through increasing susceptibility to bacterial infection [16]. In a study by Ekregbesi et al. [7], heme did not correlate with HO-1 in patients with sepsis. However, HO-1 was positively associated with the SOFA score, and patients with high HO-1 levels were found to have low survival rates. HO-1 is a rate-limiting enzyme that degrades heme during inflammation and hypoxia and may increase in abundance during sepsis and in patients with COVID-19. Therefore, in our study, HO-1 was found to be more likely than heme to increase during the development of sepsis in patients with COVID-19. Ferritin is an antioxidant involved in heme catabolism. In a meta-analysis by Kaushal et al. ferritin levels were found to be higher in patients with severe-to-critical COVID-19 or in a non-survival COVID-19 subgroup than in those with COVID-19 of lesser severity [17]. Ferritin is an important predictive factor for COVID-19 severity and needs to be adjusted for when analyzing sepsis development in patients with COVID-19. We found that HO-1 was significantly associated with 48-h sepsis development when compared with ferritin in patients with COVID-19. Bilirubin is a conventional liver dysfunction parameter for SOFA score calculation in sepsis [11]. Although bilirubin is produced from heme catabolism, it is more important as a predictor of hepatocyte cholestasis during sepsis [6].

Risk factors for sepsis and septic shock were reported to be liver disease, cardiovascular insufficiency, thrombocytopenia, and multiple sources of infection in a multicenter prospective study [18]. Therefore, to investigate the association between HO-1 and sepsis, we excluded patients with bacterial co-infection within 48 h of COVID-19 admission. Old age, male sex, underlying comorbidities, and elevated lactate dehydrogenase, C-reactive protein, and ferritin levels are established risk factors in relation to disease severity in COVID-19 [19]. In our study, after adjusting for other risk factors for sepsis in relation to COVID-19, CURB-65, d-dimer, and HO-1 remained significant risk factors, and albumin was a protective factor for sepsis. COVID-19-related pneumonia is a form of community acquired pneumonia, and CURB-65 was found to be a significant prognostic factor for COVID-19 severity [20]. In our study, CURB-65 remained a strong risk factor for sepsis development after adjusting for HO-1 analysis. Campbell et al. compared coagulopathy findings between those with COVID-19 and sepsis in 70 patients and found that plasminogen activator inhibitor 1 (PAI-1) levels were higher and d-dimer levels were lower in the plasma of patients with COVID-19 than in those with sepsis, and concluded that PAI-1 might decrease plasmin degradation and inhibit d-dimer release. In our study, patients with sepsis had higher d-dimer levels than those without sepsis, but the complicated relationship between COVID-19 and sepsis renders d-dimer insufficiently accurate to predict sepsis in COVID-19 when compared with HO-1. A low albumin level detected at the emergency department was found to be a potential marker to predict severe infection and mortality at 30 days in patients with COVID-19 [21]. Similarly, higher albumin levels were shown to be a protective factor for sepsis development within 48 h of admission in our study and would need to be adjusted accordingly.

In this study, high HO-1 level indicated a worse severity in patients with sepsis but was not related to in-hospital mortality after adjusting for other important prognostic factors, such as CURB-65, CCI, and 48-h SOFA score. HO-1 induction inhibits SARS-CoV-2 replication directly or indirectly through enhancing type 1 interferon [22]. HO-1 induction and subsequent production of carbon monoxide, bilirubin, and Fe^2+^ also have a protective effect on the inhibition of cytokine secretion and fibrin-rich clot formation. Hemin, an HO-1 inducer, has been shown to promote HO-1 overexpression and effectively suppress SARS-CoV-2 replication in cell culture studies [23]. In contrast, in a study of later stage sepsis in a cecal ligation and puncture animal model, ZnPP, an HO-1 inhibitor, was shown to improve survival and bacterial clearance in septic mice [24]. In future studies, induction therapy of HO-1 natural compounds may be provided in the early stage of sepsis for patients with COVID-19 rather than at a later stage.

This study has some limitations as a single-center, retrospective, observational study. First, a monotone likelihood limitation is shown in Table 4. Moreover, the OR of the initial CURB-65 was inflated due to the small number of samples [25]. Further multicenter prospective studies on HO-1 prediction of sepsis development are needed. Second, nuclear factor erythroid 2-related factor (Nrf2) has cytoprotective effects by regulating the expression of antioxidant and anti-inflammation genes, including the HO-1 gene [26]. This suggests that the upstream antioxidant effect should be investigated in combination with HO-1 in future to determine whether Nrf2 or HO-1 is a key factor for sepsis development in patients with COVID-19. Third, data on HO-1 levels in the late stage of sepsis were not available. Further studies to detect HO-1 levels in the early and late stages of sepsis for patients with COVID-19 are needed to test the etiopathogenetic hypothesis that HO-1 changes influence sepsis prognosis.

## Conclusions

High HO-1 levels were associated with 48-h development of sepsis after COVID-19 admission. However, HO-1 was not associated with COVID-19 mortality after adjusting for traditional risk factors. HO-1 could be a potential predictor in combination with other risk factors for early sepsis; however, further clinical multicenter prospective studies are needed to confirm its protective effect and the potential benefits of HO-1 antioxidant therapy.

## Data Availability

The data that support the findings of this study are available from Taipei Tzu Chi Hospital, Buddhist Tzu Chi Medical Foundation. Restrictions may apply to the availability of the data used under the license of the current study since they are not publicly available. However, data are available from the authors upon reasonable request and with permission from Taipei Tzu Chi Hospital, Buddhist Tzu Chi Medical Foundation.

## Abbreviations

aHR: Adjusted hazard ration
aOR: Adjusted odds ratio
aPTT: Activated partial thromboplastin clotting time
BMI: Body mass index
BUN: Blood urea nitrogen
CCI: Charlson comorbidity index
CI: Confidence interval
COVID-19: Coronavirus disease
CRP: C-reactive protein
CtV: Cycle threshold value
CURB-65: Confusion, blood urea nitrogen, respiration, blood pressure in those aged > 65 years
ELISA: Enzyme-linked immunosorbent assay
GCS: Glasgow Coma Scale
HO-1: Heme oxygenase one
MAP: Mean arterial pressure
Nrf2: Nuclear factor erythroid 2-related factor
PAI-1: Plasminogen activator inhibitor 1
qSOFA: Quick sepsis-related organ failure assessment
RT: Room temperature
RT-PCR: Reverse transcriptase polymerase chain reaction
SARS-CoV-2: Severe acute respiratory syndrome coronavirus 2
SBP: Systolic blood pressure
SOFA: Sequential Organ Failure Assessment Score
SpO_2_: Oxygen saturation of pulse oximeter
